# Genomic breeding value prediction and QTL mapping of QTLMAS2011 data using Bayesian and GBLUP methods

**DOI:** 10.1186/1753-6561-6-S2-S7

**Published:** 2012-05-21

**Authors:** Jian Zeng, Marcin Pszczola, Anna Wolc, Tomasz Strabel, Rohan L Fernando, Dorian J Garrick, Jack CM Dekkers

**Affiliations:** 1 Department of Animal Science and Center for Integrated Animal Genomics, Iowa State University, Ames, USA; 2 Department of Genetics and Animal Breeding, Poznan University of Life Sciences, Poznan, Poland; 3 Animal Breeding and Genomics Centre, Wageningen UR Livestock Research, Lelystad, The Netherlands

## Abstract

**Background:**

The goal of this study was to apply Bayesian and GBLUP methods to predict genomic breeding values (GEBV), map QTL positions and explore the genetic architecture of the trait simulated for the 15^th ^QTL-MAS workshop.

**Methods:**

Three methods with models considering dominance and epistasis inheritances were used to fit the data: (i) BayesB with a proportion π = 0.995 of SNPs assumed to have no effect, (ii) BayesCπ, where π is considered as unknown, and (iii) GBLUP, which directly fits animal genetic effects using a genomic relationship matrix.

**Results:**

BayesB, BayesCπ and GBLUP with various fitted models detected 6, 5, and 4 out of 8 simulated QTL, respectively. All five additive QTL were detected by Bayesian methods. When two QTL were in either coupling or repulsion phase, GBLUP only detected one of them and missed the other. In addition, GBLUP yielded more false positives. One imprinted QTL was detected by BayesB and GBLUP despite that only additive gene action was assumed. This QTL was missed by BayesCπ. None of the methods found two simulated additive-by-additive epistatic QTL. Variance components estimation correctly detected no evidence for dominance gene-action. Bayesian methods predicted additive genetic merit more accurately than GBLUP, and similar accuracies were observed between BayesB and BayesCπ.

**Conclusions:**

Bayesian methods and GBLUP mapped QTL to similar chromosome regions but Bayesian methods gave fewer false positives. Bayesian methods can be superior to GBLUP in GEBV prediction when genomic architecture is unknown.

## Background

Bayesian methods and the genomic BLUP procedure (GBLUP) can be used for prediction of genomic estimated breeding values (GEBV) and quantitative trait loci (QTL) detection. BayesB generally performs slightly better than GBLUP, especially when non-additive gene actions are involved [1]. Apart from Bayesian methods, GBLUP solutions can also be used to estimate marker effects [2]. The objectives of this study were 1) to identify the positions of QTL affecting the trait simulated for the 15th QTL-MAS Workshop and estimate their effects using Bayesian methods and GBLUP, 2) to explore the genetic architecture of the trait, especially regarding presence of dominance and epistasis, and 3) to predict GEBV of the individuals without phenotypes.

## Methods

### Data

The simulated population included 20 sires, 10 dams per sire and 15 full-sib progeny per dam. The genome consisted of 5 chromosomes of 1 Morgan and 1,998 evenly spaced SNPs. Sources of information for analysis included 2 generations of pedigree, genotypes for all individuals and phenotypic records for 10 progeny per family. More detailed description of the dataset is available at [3].

### Methods to predict GEBV

For additive gene-action, the statistical models BayesB [4] with π = 0.995, BayesCπ [5] and GBLUP (G1) with relationship matrix created according to [6] were applied. To examine dominance gene-action, a both additive and dominance SNP effects were fitted for every locus using BayesCπ:

$$y_{i}=\mu +\sum_{j=1}^{k}\left(X_{ij}a_{j}+W_{ij}d_{j}\right)+e_{i}$$

where *X_ij _*is the copy number of a given allele of animal *i *at SNP *j*, *W_ij _*is the dummy variable indicating whether the genotype for SNP *j *of animal *i *is heterozygous, *a_j _*(additive effect) is half the difference between homozygotes for SNP *j*, and *d_j _*(dominance effect) is the difference between heterozygote and the mean of homozygotes for SNP *j*. The priors for *a_j _*and *d_j _*were mixtures of normals as described in [5], with effect specific values for π (π*_a _*and π*_d_*) and variance σ^2 ^($\sigma_{a}^{2}$and $\sigma_{d}^{2}$). Gibbs sampling was used to sample the posterior distribution of model parameters. SNP effects were estimated by the mean of the sampled values. GEBVs were predicted as the linear combination of the SNP substitution effects. *GenSel *[7] was used to implement the Bayesian methods.

In GBLUP the presence of dominance was investigated using a model with an additional random dominance effect (G2) for each animal. The variance-covariance matrix for this effect was created similar to the genomic relationship matrix **G**, except genotypes were coded as 1 for heterozygotes and 0 for both homozygotes. The third model (G3) had an additional random additive-by-additive epistatic effect for each animal, with **G^2 ^**as the variance-covariance matrix. GEBV were estimated using models G1 to G3 with variance components estimated using *ASReml *[8].

### Methods to map QTL

In the Bayesian methods, QTL positions were identified based on the absolute value of estimated SNP effects, the posterior inclusion probability (or model frequency) for each SNP, and the variance of GEBV (or window variance) for any 10 consecutive SNP standardized by dividing by the total variance of GEBV in the population. The QTL were mapped to the SNP that explained the largest proportion of the total variance of GEBV within the significant overlapping windows, whose variances were in top $\left(1-\hat{\pi }\right)\times 100\%$ in BayesCπ or visually remarkably higher than the background window variances in BayesB. In GBLUP model G1, allele substitution effects were estimated following [2]:

$$\alpha =\sigma_{\alpha }^{2}Z^{\prime }G^{-1}\hat{a}$$

where **α **is the vector of allele substitution effects, $\sigma_{\alpha }^{2}=\sigma_{a}^{2}/2\sum {\text{p}}_{\text{i}}\left(1-{\text{p}}_{\text{i}}\right)$ where $\sigma_{\text{a}}^{2}$ is additive genetic variance, **Z **is the genotype matrix with dimensions equal to the number of individuals by the number of SNPs, and **â **is the vector of GEBV obtained from GBLUP. Given the estimated SNP effects, QTL were mapped to the positions where the SNP had visually significant effects on the trait.

## Results

### Estimated variance components

Table 1 shows the estimated variance components for each method. All models, especially GBLUP, slightly underestimated the both genetic and environmental variance components. Heritabilities from the Bayesian methods were close to the true heritability. The dominance models under BayesCπ and GBLUP gave negligible estimates of dominance genetic variance. No epistatic variance was detected.

**Table 1:** Estimated variance components and heritability (h^2^)

Methods	Genetic Variance Components			Residual	Total	h^2^
	
	Additive	Epistasis	Dominance			
True Value	26.35			61.49	87.84	0.3

BayesB	24.61	-	-	60.17	84.78	0.29

BayesCπ						

AM	24.19	-	-	60.29	84.48	0.286

DM	24.27	-	0.12	60.16	84.55	0.287

GBLUP						

G1	22.09	-	-	59.8	81.89	0.269

G2	22.19	-	0.51	59.34	82.03	0.27

G3	22.09	6.20E-06	-	59.8	81.89	0.269

Obtained by BayesB (π = 0.995), BayesCπ using an additive model (AM) and dominance model (DM), GBLUP using additive model (G1), with additional random dominance effects (G2) and epistatic effects (G3).

### QTL mapping

**Figure 1:**
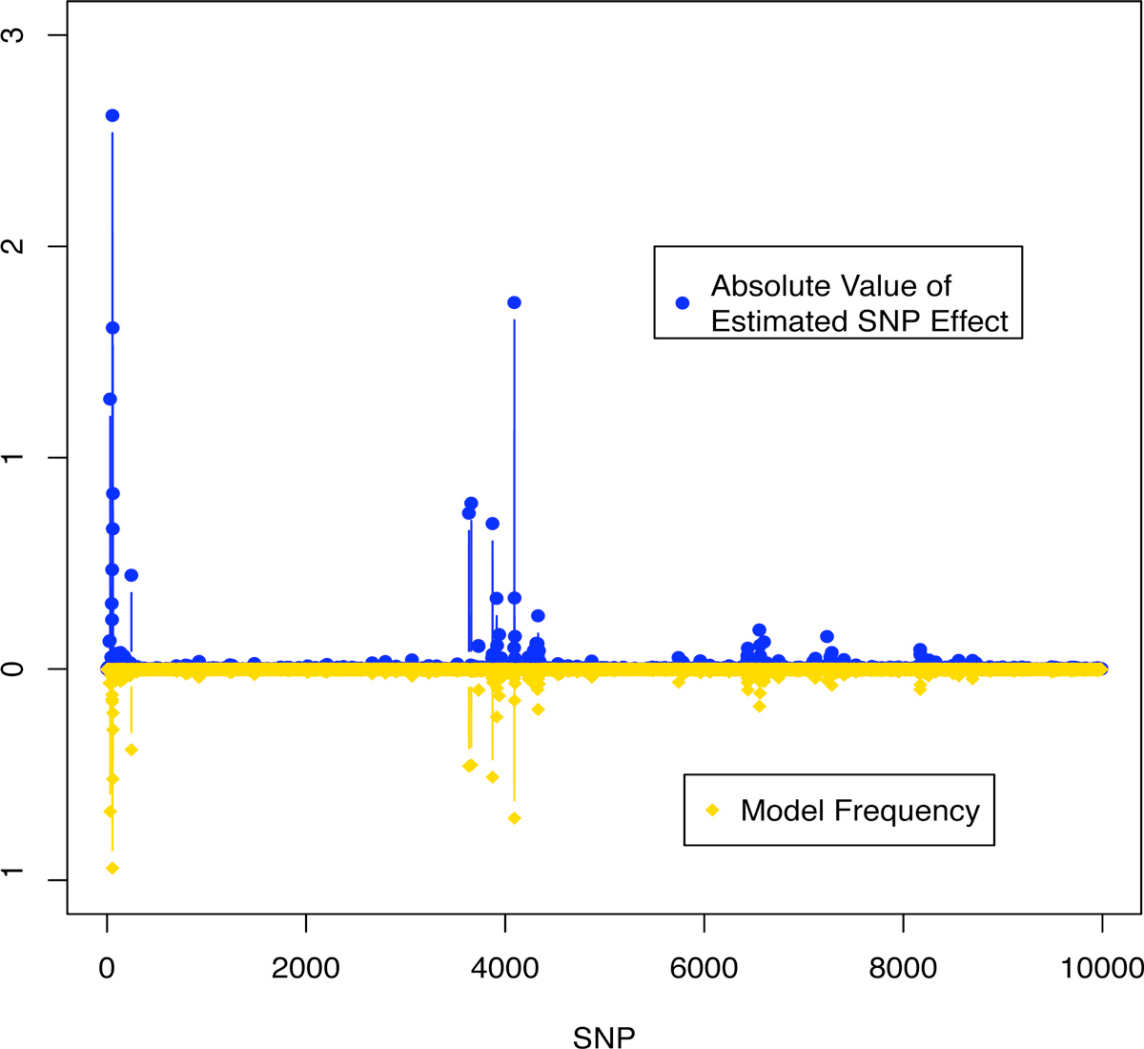
**Single SNP association signals across the genome**. Absolute value of estimated SNP effects and model frequencies obtained by BayesCπ using an additive model.

**Figure 2:**
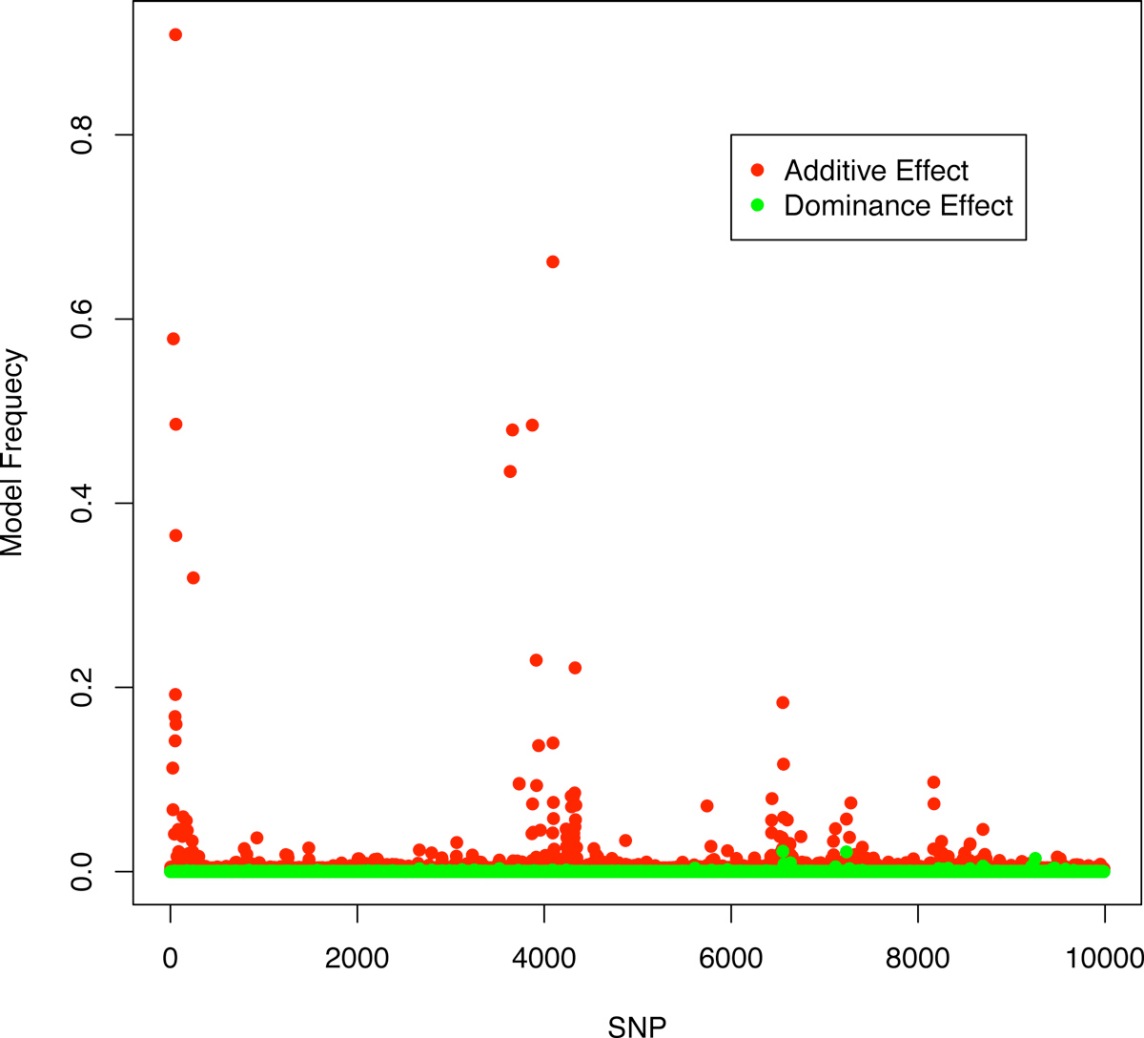
**Model frequencies of SNPs across the genome**. For additive and dominance effects obtained by BayesCπ using a dominance model.

**Figure 3:**
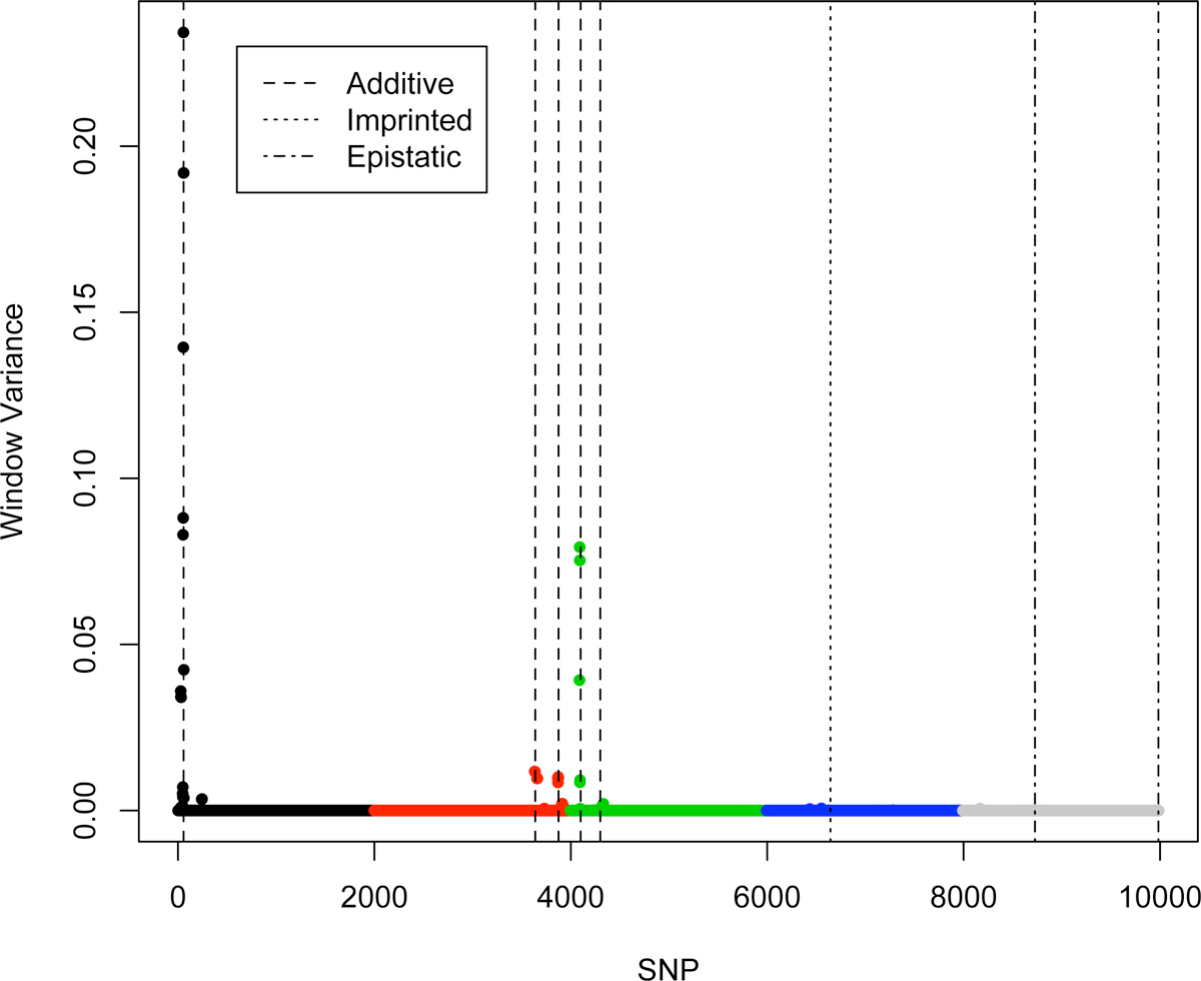
**10-SNP window variances across the genome obtained by BayesCπ**. Colours differentiate chromosomes and vertical lines indicate true simulated QTL locations along with their gene actions.

**Figure 4:**
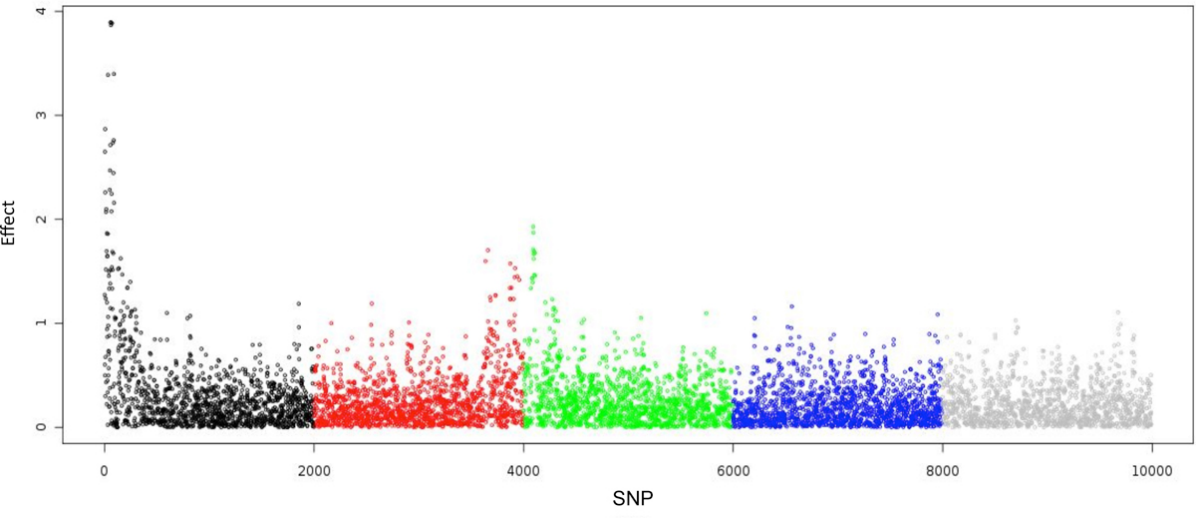
**Estimated marker effects (absolute values) across the genome obtained by GBLUP**. Colours differentiate chromosomes.

Figure 1 shows the estimated SNP effects and single SNP model frequencies for BayesCπ with the additive model. Two regions showed strong evidence of association, indicating QTL. The additive signals of SNP from the dominance model of BayesCπ shown in Figure 2 confirm the results of the additive model and suggest the absence of dominance. The top 10-SNP window variances were markedly higher than the background window variances (Figure 3). While the top 10-SNP window variances agreed with the significant regions found by single SNP signals (Figure 2), the moderate single SNP signals towards the end of the genome were absent in the window variances. BayesB gave similar results to BayesCπ thus the results are not shown. However, the selection of significant windows in BayesB was more subjective. GBLUP resulted in more signals and larger noise, which increased the probability of false positives (Figure 4). It turned out that BayesB detected 6 QTL, BayesCπ 5 and GBLUP 4, out of 8 simulated QTL regions. Except for one false positive on chromosome 1, all QTL identified by BayesB and BayesCπ were in the true simulated QTL regions. Under the additive model, a QTL region on chromosome 4 was successfully detected by BayesB, and at the cost of some false positives by GBLUP, but missed by BayesCπ. This QTL, however, turned out to be an imprinted QTL. None of the methods found the two simulated epistatic QTL on chromosome 5.

### Predictive accuracy of GEBV

Table 2 shows correlations between GEBV for validation individuals from different methods. Compared with the true simulated breeding values, BayesCπ gave the highest accuracy of 0.939, which was slightly higher than BayesB (0.934). GBLUP gave the lowest accuracy (0.825). Correlations between GEBV from BayesB and BayesCπ were close to 1.

**Table 2:** Correlations among GEBV

Method	BayesB	BayesCπ	GBLUP
BayesCπ	0.997		

GBLUP	0.918	0.897	

TBV	0.934	0.939	0.825

Obtained by Bayesian methods and GBLUP, and with simulated true breeding values (TBV) for validation individuals.

## Discussion

The simulated trait was affected by one QTL with major and seven with minor effects. Two QTL were interacting with each other (epistasis) and one was imprinted. All approaches detected the major QTL and three to six QTL with smaller effects. The Bayesian methods detected more simulated QTL regions and gave fewer false positives than GBLUP. GBLUP failed to find one of the two QTL that were close to each other. This confirms the finding of [8] that when the genetic architecture of the trait is complex, Bayesian methods are superior to GBLUP in QTL mapping.

The failure to detect the imprinted QTL for BayesCπ and the epistatic QTL for BayesB and BayesCπ reveals some drawbacks of basing QTL mapping solely on window variances. A 10-SNP window may include too much noise, which results in shrinkage of the signals towards zero. Thus, the variance of the causative region may be underestimated. As shown in Figures 1 and 2, although some single SNP signals were shown for the imprinted and epistatic QTL on chromosome 4 and 5, the small window variances prevented these regions from being considered significant (Figure 3). For the major QTL, 10-SNP windows may be too narrow to cover the entire causative region, which resulted in two QTL being identified. Moreover, if the parental origins of alleles were known, an additive model that fits substitution effects of the alleles specific to their parental origins, or a dominance model that fits dominance effects specific to the type of heterozygotes (01 or 10) is expected to capture the imprinting inheritance.

GEBV obtained using Bayesian and GBLUP analyses were highly correlated among each other, which agrees with [10]. In accord with earlier QTL-MAS workshops [1,11], Bayesian methods yielded higher accuracy of GEBV (0.93-0.94) than GBLUP (0.83). Because most SNP had no effects on the trait, including spurious SNP in the model introduced noise to GEBV and impaired the predictive accuracy. For high-density SNP panels or DNA sequencing data, Bayesian models are considered more robust and the superiority over GBLUP is expected to increase.

## Conclusions

Bayesian methods and GBLUP revealed the additive genetic attributes of the simulated trait. The number of indicated regions and their positions were in good agreement with the truth. Bayesian methods were superior to GBLUP in QTL mapping, with fewer false positives. The window variance is a plausible criterion to identify QTL using Bayesian methods, although some drawbacks exist. The mutual correlations among alternative methods were close to one but Bayesian methods yielded higher accuracy for GEBV than GBLUP.

## List of abbreviations used

QTL: quantitative trait locus; BLUP: best linear unbiased prediction; GBLUP: BLUP with a realized relationship matrix; TABLUP: BLUP with a trait specific relationship matrix; GEBV(s): genomic estimated breeding value(s); TBV(s): true breeding value(s); SNP: single nucleotide polymorphism.

## Competing interests

The authors declare that they have no competing interests.

## Authors' contributions

MP and JZ drafted the paper. JZ, MP and AW performed the analyses. TS, AW, RF, DG, JD critically revised the manuscript and mentored the analyses. All authors read and approved the manuscript.
